# Abrupt increased serum creatinine in a hyperferritinemia patient treated with deferoxamine after cord blood transplantation: a case report with literature review

**DOI:** 10.1186/s40780-023-00287-w

**Published:** 2023-06-01

**Authors:** Hirokazu Nakayama, Yoshimasa Kamoda, Michiya Tanuma, Toshiaki Kato, Kensuke Usuki

**Affiliations:** 1 Department of Pharmacy, NTT Medical Center Tokyo, 5-9-22 Higashi-gotanda, Shinagawa-ku, Tokyo, 141-8625 Japan; 2 Department of Hematology, NTT Medical Center Tokyo, Tokyo, Japan

**Keywords:** Deferoxamine, Colistin, Acute kidney injury, Cord blood transplantation

## Abstract

**Background:**

Erythrocyte transfusion is an indispensable component of supportive care after hematopoietic stem cell transplantation (HSCT). However, HSCT recipients are susceptible to the development of acute kidney injury (AKI) with multifactorial causes. We report a case of a rapid elevation in serum creatinine associated with deferoxamine after cord blood transplantation (CBT).

**Case presentation:**

A 36-year-old Japanese male diagnosed with relapsed Philadelphia-positive acute lymphoblastic leukemia received CBT. At day 88 post-CBT, multidrug-resistant *Pseudomonas aeruginosa* (MDRP) was isolated from urine culture. Subsequently, colistin 200 mg/day was administered parenterally for treatment of epididymitis from day 91 to 117 post-CBT. Despite concomitant administration of potential nephrotoxic agents such as piperacillin-tazobactam, acyclovir, and liposomal amphotericin B, no development of AKI was observed during this period. At day 127 post-CBT, MDRP was detected in blood and urine cultures, and colistin 200 mg/day was re-started parenterally. Due to extremely higher ferritin level, deferoxamine was administered intravenously at day 133 post-CBT. While serum creatinine was 1.03 mg/dL before starting deferoxamine, the level increased to 1.36 mg/dL one day after commencing deferoxamine (day 134 post-CBT), and further increased to 2.11 mg/dL at day 141. Even though colistin was discontinued at day 141 post-CBT, serum creatinine continued to increase. Deferoxamine was withdrawn at day 145 post-CBT, when serum creatinine peaked at 2.70 mg/dL. In addition, no cylinduria is observed during the period of development of AKI. In adverse drug reaction (ADR) assessment using Naranjo probability score, the scores of 3 in deferoxamine and 2 in colistin, respectively, indicated “possible” ADR. However, while colistin-associated AKI manifested early onset, recovery time within 2 weeks after discontinuation and development of cylinduria, this case was discordant with the properties. Furthermore, in the literature review, development of AKI within 1 day, including sudden increase in serum creatinine or abrupt reduction in urine volume, was reported in 3 identified cases.

**Conclusions:**

We considered the rapid creatinine elevation to be the result of deferoxamine rather than ADR caused by colistin. Therefore, careful monitoring of kidney function is required in recipients of HSCT treated with deferoxamine.

## Background

Erythrocyte transfusion is an indispensable component of supportive care after hematopoietic stem cell transplantation (HSCT). In the post-transplant period, multiple blood transfusions primarily cause iron overload, potentially leading to severe infection, hepatic dysfunction and heart failure, and increasing the risk of non-relapse mortality after HSCT [1–3]. In these patients, iron chelation therapy such as deferoxamine is relatively safe and efficacious to reduce total body iron burden, and is beneficial in improving complication after transplantation [3]. Furthermore, HSCT recipients are susceptible to develop acute kidney injury (AKI) with multifactorial causes including critical illness, nephrotoxic medication, infection and graft versus host disease [4].

Nosocomial infection caused by multidrug-resistant (MDR) microorganisms in patients who have undergone HSCT remains a significant issue [5]. While colistin, a polymyxin antimicrobial agent, is used to treat MDR Gram-negative bacteria infection, a major concern with colistin use is the occurrence of AKI as an adverse effect [6].

In patients with critically ill, N-acetylcysteine in combination with deferoxamine led to reduced serum creatinine [7]. In contrast, a case of AKI associated with deferoxamine treatment in a renal transplant patient has been reported [8] Thus, there is a paucity of information on the safety of deferoxamine treatment after HSCT. We report a case of a rapid elevation in serum creatinine associated with deferoxamine after cord blood transplantation (CBT).

## Methods

A literature review of deferoxamine-associated AKI was conducted using the PubMed database from 1966 to February 2023. The medical subject headings (Mesh) were used in this search. The search formula consisted of “deferoxamine” [Mesh] AND (“renal insufficiency” [Mesh] OR “creatinine” [Mesh] OR “nephrotoxicity” [All Fields]). The search was limited to articles, including case reports and case series studies, published in English. Studies were included if the changes of serum creatinine and/or urine volume for kidney function were assessed. Articles with all potentially relevant articles based upon titles and abstracts were initially retrieved. Subsequently, the review was completed by reading the full texts of selected articles met the study criteria by two researchers.

In the analysis of causal relationships in this case, the adverse drug reaction (ADR) probability of (i) deferoxamine and (ii) colistin was evaluated using the ADR (Naranjo) probability scale [9]. In this analysis, AKI was defined as (i) an increase of 50% or more from baseline serum creatinine value or (ii) less than 0.5mL/kg/hr of urine volume, according to a previous report [10]. The last search was conducted on April 30 2023.

## Case presentation

A 36-year-old Japanese male with Philadelphia-positive acute lymphoblastic leukemia. Due to relapse after allogeneic peripheral blood stem cell transplantation from his brother, he received CBT followed remission induction chemotherapy for 14 days and deferasirox 1000 mg/day for 12 days.

**Fig. 1:**
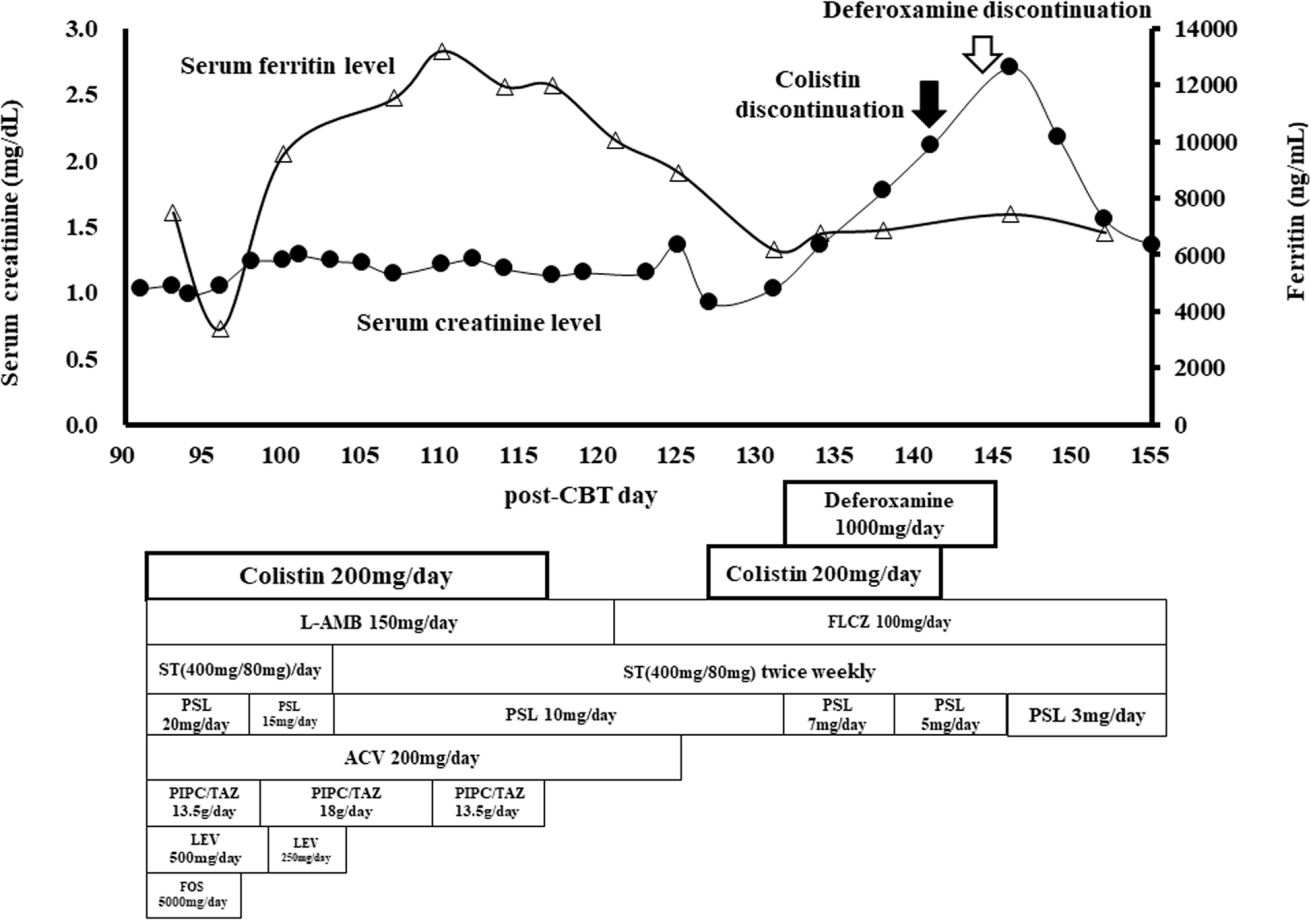
Time courses of serum creatinine and ferritin levels in relation to treatment modalities in the patient treated with deferoxamine after cord blood transplantation. Solid line with closed circles shows serum creatinine levels, and dotted line with open triangles shows serum ferritin levels. Closed arrow shows the day of discontinuation of colistin, and open arrow shows the day of discontinuation of deferoxamine. Abbreviations: ACV, acyclovir; CBT, cord blood transplantation; L-AMB, liposomal amphotericin B; FLCZ, fluconazole; FOS, foscarnet; LEV, levetiracetam; PIPC/TAZ, piperacillin-tazobactam; PSL, prednisolone; ST, sulfamethoxazole-trimethoprim

While no calcineurin inhibitor toxicity was observed, despite cyclosporine administration until day 65 post-CBT, he developed intestinal acute graft versus host disease (GVHD) grade 3 which was ameliorated at day 176 post-CBT, following parenteral methylprednisolone, thereafter oral prednisolone. At day 88 post-CBT, multidrug-resistant *Pseudomonas aeruginosa* (MDRP) was isolated from urine culture. Subsequently, colistin 200 mg/day was administered parenterally for treatment of epididymitis from day 91 to 117 post-CBT. Despite concomitant potential nephrotoxic agents administered piperacillin-tazobactam, acyclovir and liposomal amphotericin B, a slight increase in serum creatinine, +0.30 mg/dL, was observed between during this period (Fig. 1).

At day 122 post-CBT, body temperature was elevated to 40.0°C, and meropenem 2 g/day was given until day 126 post-CBT. At day 127 post-CBT, MDRP was detected in blood and urine cultures, and colistin 200 mg/day was re-strated parenterally, concomitant with another potential nephrotoxic agent fluconazole 100 mg/day, resulting in amelioration of MDRP infection. In addition, concomitant sulfamethoxazole/trimethoprim 400 mg/80 mg daily was administrated until day 106 post-CBT and thereafter the dose was reduced to 400 mg/80 mg twice weekly.

A total of 240 units of erythrocytes were transfused between day 394 pre-CBT and day 132 post-CBT due to delayed engraftment. At day 133 post-CBT, deferoxamine 1000 mg/day was started to avoid excess iron overload, because ferritin level fluctuated between 6184 and 13198 ng/mL. Due to difficulty in receiving an oral chelate agent, deferoxamine was administered intravenously; a daily dose of 1000 mg was diluted in 100 mL normal saline and infused for 2 hours. Doses of colistin and deferoxamine were decided considering creatinine clearance (62 mL/min) based upon body weight (43 kg), respectively. While serum creatinine was 1.03 mg/dL before starting deferoxamine, the level abruptly increased to 1.36 mg/dL at day 134 post-CBT, one day after commencing deferoxamine, and further increased to 2.11 mg/dL at day 141 post-CBT. Despite discontinuation of colistin at day 141 post-CBT, serum creatinine continued to increase (Fig. 1). Serum creatinine peaked at 2.70 mg/dL at day 146 post-CBT. Deferoxamine was, eventually, administered until day 145 post-CBT. Thereafter, kidney function recovered to baseline level at day 163 post-CBT, 18 days after discontinuation of deferoxamine. In addition, cylinduria is not observed at day 131-post CBT and 146-post CBT. Thereafter, while the treatment of erythrocytes transfusion was continued until day 384 post-CBT, no implementation of iron-chelate therapy was performed until day 246 post-CBT. However, due to hyperferritinemia, deferasirox was readministrated between day 247 and 447 post-CBT. During the period of deferasirox therapy, no increase in serum creatinine levels was observed.

On the Naranjo probability scale, the scores of 3 in deferoxamine and 2 in colistin, respectively, indicated “possible” ADR (Table 1).

**Table 1:** Classification of Adverse Drug Reactions Probability for Deferoxamine and Colistin Assessed by the Naranjo Probability Scale [9]

Number	Questions	Outcome of DFO and colistin	**DFO Score**	**Colistin score**
1	Are there previous conclusive reports of this reaction?	AKI is described in package insert of DFO. Colistin induced AKI is most prominent.	**+1**	**+1**
2	Did ADR appear after the drug was given?	AKI developed after administration of DFO and colistin, respectively	**+2**	**+2**
3	Did ADR improve when the drug was discontinued or a specific antagonist was given?	AKI was ameliorated after discontinuation of DFO. S-Cre was continuously increased despite cessation of colistin.	**+1**	**0**
4	Did ADR reappear upon re-administering the drug?	Neither drug was re-administrated.	**0**	**0**
5	Were there other possible causes for the reaction?	Due to absence of clinical conditions, AKI caused by DFO or colistin was considered.	**-1**	**-1**
6	Did ADR reappear upon administration of placebo?	Not done for either	**0**	**0**
7	Was the drug detected in the blood or other fluids in toxic concentrations?	Not analyzed in either	**0**	**0**
8	Was the reaction worsened/lessened upon increasing/decreasing the dose?	Not applied, due to no dose adjustment for either	**0**	**0**
9	Did the patient have a similar ADR to the drug or a related agent in the past?	No previous exposure for either	**0**	**0**
10	Was ADR confirmed by any other objective evidence?	Not done for either	**0**	**0**
	**Total score**		**3**	**2**

Total scores range from -4 to +13. Definite, ≥ 9; Probable, 5‒8; Possible, 1‒4; Doubtful, ≤ 0. Scores for this case are shown in bold numeric characters

*Abbreviations:* *ADR* Adverse drug reaction, *AKI* Acute kidney injury, *DFO* Deferoxamine, *S-Cre* Serum creatinine

## Discussion and conclusion

To the best of our knowledge, this is the first case report suggesting that an abrupt increase in serum creatinine was associated with deferoxamine during the period of colistin therapy after CBT. It remains unclear whether deferoxamine is indicated for hyperferritinemia within the first few months after CBT. In addition, the frequency of deferoxamine-associated AKI is unclear. Deferoxamine infusion as chelation treatment is considered particularly useful for hyperferritinemia patients after HSCT due to significant reduction of total body iron burden [11]. In the present case, intravenous deferoxamine was administered to avoid continuous excess iron overload, with ferritin levels fluctuating between approximately 6000 and 13000 ng/mL during peri-CBT period.

A total of 217 records was identified through the database. Based upon inclusion criteria, 4 case report and 2 case series were eligible. As shown in Table 2, deferoxamine has been associated with AKI in 10 cases, including adult aged ≥ 18 years in 7 and less than 18 years in 3, in individual case reports and small case series studies [7, 12–16]. Eight of 10 cases developed AKI with increase in serum creatinine more than 2 folds compared with baseline value or reduction of urine volume less than 0.5mL/kg/hr. Development of AKI within 24 hours was reported in 3 identified cases [12, 13, 16]. In the 3 cases, sudden increases in serum creatinine were caused by accidental administration of high dose deferoxamine.

**Table 2:** Literature review of intravenous deferoxamine associated acute kidney injury

**Case**	**Authors (year)**	**Age (years)/Gender**	**Main Diagnosis**	**Baseline serum creatinine (mg/dL)**	**Maximal serum creatinine (mg/dL)**	**AKI onset time**	**Daily dose (Infusion rate)**^**a)**^	**Ferritine level (ng/mL)**^**b)**^	**Outcome**
1	Clajus et al (2008) [7]	58/M	Goodpasture’s syndrome ^c)^	1.4	2.8	21 days	2g (2.4mg/kg/hr)	1747	Recovery at 2 weeks after cessation
2	Prasannan (2003) [12]	17/M	Sickle cell-beta thalassemia	0.4	2.2	Within hours ^d)^	45g (87.5mg/kg/hr)	8000	Recovery after hemodialysis
3	Cianciulli (1992) [13]	19/M	Thalassemia	NA ^e)^	6.1	18 hours ^d)^	33.7g (39mg/kg/hr)	7000	Recovery after hemodialysis
4	Batey et al (1979) [14]	14/M	Thalassemia	NA	NA ^f)^	10 days	3g (NA)	14000	Death
5	Cartei et al (1975) [15]	59/M	Polycythemia rubra vera	1.0	2.0	15 days	3g (NA)^g)^	NA	Recovery after dose reduction
6	Cartei et al (1975) [15]	76/M	Sideroblastic Anemia	1.4	3.6	20 days	4g (NA)^g)^	NA	Recovery after dose reduction
7	Cartei et al (1975) [15]	67/F	Secondary Hepatopathic Hemochromatosis	0.4	1.5	NA	4g (NA)^g)^	NA	Recovery after dose reduction
8	Koren et al (1989) [16]	21/M	Thalassemia	1.1	3	11 days	12.1g (10mg/kg/hr)	5300	Recovery at 5 days after cessation
9	Koren et al (1989) [16]	18/M	Thalassemia	0.8	1.2	10 days	10.3g (10mg/kg/hr)	14000	Recovery at 1 month after cessation
10	Koren et al (1989) [16]	2/F	Ingestion of ferrous fumarate tables	0.4	2.8	1 day	NA (10mg/kg/hr)	284	Recovery at 2 weeks after cessation

In cases 2 and 3, deferoxamine was administrated due to mis-programmed infusion pump. In case 1, Cyclosporine A and furosemide, potential nephrotoxic agents, were concomitantly administrated. In case 4, furosemide, a potential nephrotoxic agent, was concomitantly administrated. In other cases, concomitant agents were not available

*Abbreviations:* *AKI* Acute kidney injury, *hr* Hour, *NA* Not available

^a^Daily dose and infusion rate were shown as the value at the development of AKI

^b^The value at the commencement of deferoxamine therapy

^c^This patient received cadaver kidney transplantation.

^d^AKI occurrence time after accidental deferoxamine administration

^e^While kidney function was controlled, no details were available

^f^Oliguric renal failure was considered since urine volume was reduced from 1500 mL/24hr to 200mL/24hr

^g^First 1g was administrated as intramuscular injection, and thereafter as slow infusion

In this case, AKI developed during the period of treatment for GVHD. Indeed, GVHD is suggested as a common risk factor for AKI after HSCT [17, 18]. In this regard, treatment with steroid was continued, resulting in the amelioration of GVHD. Consequently, no symptoms of dehydration due to diarrhea caused by GVHD were observed during the period of deferoxamine administration.

In ADR assessment using Naranjo probability scale, the scores of deferoxamine and colistin indicated “possible” ADR, respectively. Therefore, no definitive conclusion has been reached. In this regard, since idiosyncratic ADRs would not have high scores indicating a definite or probable ADR using the Naranjo scale, it has not been validated for use in patients with critical illness or suffering specific organ toxicity [19]. In addition, the clinical manifestations of colistin-associated nephrotoxicity are the following; occurrence within the first 5 days of colistin treatment, recovery in serum creatinine within 1 to 2 weeks after the discontinuation in moderate AKI, and development of cylinduria [20–23]. However, this case was discordant with these properties. Furthermore, the following various risk factors of colistin-associated nephrotoxicity were suggested; dose ≥5 mg/kg/day, length of colistin treatment, receiving concomitant multiple nephrotoxic agents, age, gender, hypoalbuminemia less than 2.0 or 3.2 g/dL, hyperbilirubinemia more than 5 mg/dL in total-bilirubin, and severity of the illness. [6, 23, 24]**.** In this regard, while the length of colistin exposure and gender were relevant in this case, the duration of colistin therapy included an interruption of 10 days. Therefore, we consider the length of exposure to have had less influence. By contrast, in deferoxamine-associated AKI, the duration required for recovery to the baseline value in serum creatinine was 2 weeks to 1 month [16]. The time course in this case was concordant with the report. In addition, plausibility is an indispensable criterion in assessing the causal relationships [25, 26]. For the plausibility of development of AKI, we considered the rapid creatinine elevation to be a result of administration of deferoxamine rather than colistin.

While the approved infusion rate of deferoxamine was less than 15 mg/kg/hr, the standard intravenous administration is 40–50 mg/kg/day over 8–12 hours in adults [27]. Deferoxamine was administrated at a high infusion rate (approximately 12 mg/kg/hr) in this case. In 5 of 10 cases with AKI, deferoxamine with infusion rate more than 10 mg/kg/hr was administrated intravenously (Table 2). Koren et al demonstrated that in an animal model, AKI was caused by the administration of deferoxamine of 5 mg/kg/hr [16]. The rapid intravenous infusion of deferoxamine may be associated with AKI. In addition, there is a limitation in the procedure of the literature review, due to the search strategy focused upon the change of creatinine and/or urine volume. Clearly, further studies of the relation between AKI and infusion rate are required.

In our case, a sudden rise in serum creatinine was observed immediately following initiation of deferoxamine. Colistin is considered to cause acute tubular necrosis due primarily to tubular epithelial cell membrane permeability [28]. Meanwhile, deferoxamine reduces renal perfusion *via* inhibition of prostanoid synthesis [29]. This synergic nephrotoxicity causing tubular damage is a possible explanation for the AKI observed in our case.

Causality was not rigorously proven due to a number of limitations of the assessment for kidney function, using only serum creatinine as well as ADR scoring. Despite the limitations, in this post-CBT patient with hyperferritinemia and MDRP, deferoxamine use may have resulted in nephrotoxicity. Therefore, careful monitoring of kidney function is required in recipients of HSCT treated with deferoxamine.

## Data Availability

The datasets generated during and/or analyzed during the current study are available from the corresponding author on reasonable request.

## Abbreviations

ADR: Adverse drug reaction
AKI: Acute kidney injury
CBT: Cord blood transplantation
GVHD: Graft versus host disease
HSCT: Hematopoietic stem cell transplantation
Mesh: Medical subject headings
MDR: Multidrug-resistant
MDRP: Multidrug-resistant *Pseudomonas aeruginosa*
